# Predictive health index: integrating body composition and heart rate variability metrics with artificial intelligence to predict chronic disease risk and specific chronic non-communicable diseases

**DOI:** 10.1007/s42000-025-00727-2

**Published:** 2025-12-08

**Authors:** Dario Boschiero, Andrea Gallotta, Francesca Ferrari, Konstantina Dragoumani, George Lamprou, Dimitrios Vlachakis, George P. Chrousos

**Affiliations:** 1 BioTekna - Biomedical Technologies, Marcon, Venice, Italy; 2 Endocrine Unit and University Research Institute of Maternal and Child Health and Precision Medicine, School of Medicine, National and Kapodistrian University of Athens, Athens, 11527 Greece; 3 Laboratory of Genetics, Department of Biotechnology, School of Applied Biology and Biotechnology, Agricultural University of Athens, Athens, 11855 Greece; 4 Centre for Craniofacial and Regenerative Biology, Faculty of Dentistry, Oral & Craniofacial Sciences, Open Academy of Medicine, King’s College London, Floor 27 Tower Wing, Guy’s Campus, London, SE1 9RT UK

**Keywords:** Predictive health, Artificial intelligence, Body composition analysis, Heart rate variability (HRV), Chronic non-communicable diseases (NCDs), Non-invasive diagnostics

## Abstract

**Purpose/objective:**

The field of predictive medicine focuses on assessing disease risk and implementing preventive strategies with a view to either preventing disease onset entirely or significantly minimizing its impact on affected individuals. An emerging subfield, predictive health, extends this approach by targeting healthy individuals, emphasizing proactive lifestyle modifications to reduce the risk or to potentially reverse the progression of chronic non-communicable diseases (NCDs).

Predictive health employs a diverse array of tools to forecast health and disease, thereby transitioning from a reactive to a proactive healthcare model. By extending the duration of good health and reducing the incidence, prevalence, and costs of NCDs, it has the potential to revolutionize medical practice. This approach redirects the focus of medicine from treating NCDs to preventing them through lifestyle modifications, marking a fundamental shift toward disease prevention and long-term well-being.

**Methods:**

This study developed and validated a Predictive Health Index (PHI) in the context of cardiovascular, metabolic, psychological, neoplastic, and chronic inflammatory diseases to assess health status and predict the risk of the disease contextually. A variety of metrics were obtained from non-invasive instrumental diagnostic tests, including body composition analysis using advanced bioimpedance techniques (BIA-ACC^®^) and heart rate variability (HRV) analysis employing a photoplethysmography (PPG) system. The data were obtained from 35,405 clinically monitored individuals over about 5 years. Artificial intelligence and a random forest machine learning algorithm were trained and tested to create the PHI.

**Results:**

The results demonstrated highly significant (p-value < 0.0001) predictive performance concerning the PHI, successfully distinguishing healthy subjects from those at high risk of disease. PHI can thus be a fast, non-invasive, easy-to-use, highly accurate tool for assessing health and predicting risk of developing NCDs.

**Conclusion:**

The acquired information could be extremely helpful for strengthening lifestyle measures and intervening early to prevent or reverse disease development.

## Introduction

While preventive medicine has always been a fundamental component of Hippocratic medicine, the advent of predictive medicine and predictive health represents a significant evolution in the modern healthcare field, with a shift towards a focus on prognostication and prevention as opposed to treatment of diseases. Predictive medicine currently aims to assess an individual’s likelihood of developing a chronic disease before clinical manifestation, utilizing various technologies and diagnostic tools. One key objective is the early identification of disease risk [1, 2]. Through genetic testing, biomarkers, and other diagnostic methods, it is possible to determine an individual’s predisposition to develop specific diseases. Additionally, predictive medicine facilitates the implementation of targeted preventive measures to mitigate disease onset or reduce its impact. Furthermore, the personalization of healthcare enables the development of individualized health plans, considering genetic profiles and other risks [3, 4].

Predictive health constitutes an emerging branch of predictive medicine that focuses on healthy and/or diseased yet high-performing individuals. The overarching objective is to proactively modify lifestyle to promote long-term wellness and mitigate the risk of chronic non-communicable diseases (NCDs). Chronic diseases such as cardiovascular disorders, metabolic syndrome, psychological conditions, neoplastic diseases, and chronic inflammatory disorders impose a significant burden on healthcare systems worldwide, accounting for most of the morbidity and mortality that plagues humanity [5–9]. These conditions are often influenced by modifiable risk factors, highlighting the critical role of early intervention and prevention in improving health outcomes [2, 10], extending life expectancy [4, 11], and reducing healthcare burden and cost [12, 13]. This approach may include continuous health monitoring using wearable devices and other technologies to monitor vital signs and other health indicators [14, 15]. Additionally, personalized lifestyle interventions are implemented, such as advice on dietary habits, physical activity, sleep patterns or stress management techniques, and other healthy lifestyle habits based on the individual’s specific needs [16, 17]. Finally, dissemination of educational material and fostering of awareness appear as essential measures, contextually promoting greater awareness of risk factors associated with various health disorders and strategies for effective management [18, 19].

Predictive health uses several approaches and instruments to predict and enhance health, including genetic testing and biomarkers that identify genetic predispositions to certain diseases and/or indicate the subclinical presence of chronic disease before symptoms appear. Predictive health thus uses genetic testing [20, 21] and several physiologic and biochemical biomarkers [22, 23] to identify genetic predispositions to certain diseases. Monitoring technologies [14, 15], such as wearable devices and mobile applications are employed to follow parameters, such as heart rate, sleep length and quality, and physical activity. Sophisticated data analyses are also used [24, 25], including advanced artificial intelligence (AI) algorithms and machine learning (ML) when necessary to analyze massive amounts of health data (BIG data) as a way of identifying predictive health patterns.

The field of predictive health has witnessed the incorporation of non-invasive diagnostic technologies and AI as its practices, resulting in the provision of precise, personalized insights into an individual’s health status and genetic information. Non-invasive approaches, such as bioimpedance analysis (BIA) and heart rate variability (HRV) monitoring with specific devices (BIA-ACC^®^, Biotekna, Marcon, Italy), (PPG Stress Flow^®^, Biotekna, Marcon, Italy) respectively, have emerged as powerful methods for capturing key physiological and autonomic nervous system parameters associated with health and disease. BIA quantifies body composition metrics, including fat mass, muscle mass, and hydration status [26–28], while HRV assesses the balance of the autonomic nervous system by analyzing heart rate fluctuations in response to changes in vagus nerve tone [29–31]. Both devices are non-invasive and have been CE-marked for diagnostic/monitoring use in the EU since 2004; BIA-ACC^®^ provides an extended panel of body composition variables, while PPG Stress Flow^®^ acquires optical pulse signals for HRV and autonomic nervous system (ANS) parameter estimation. These technologies offer a rapid, cost-effective, and patient-friendly alternative to traditional diagnostic methods, enabling continuous monitoring and early detection of subclinical abnormalities.

AI and its subbranches, particularly ML algorithms and deep learning (DL) techniques, have also revolutionized the field of data analysis in predictive health [32]. These methods facilitate the integration of complex, multidimensional datasets from data derived from any biological sample in both invasive and non-invasive ways, thus enabling the identification of patterns and relations that are not readily apparent through traditional statistical techniques. Leveraging substantial datasets, AI algorithms can yield highly precise predictions of disease risk and health status, thus facilitating timely and targeted interventions and personalizing treatment plans [32]. The exploitation of ML and DL techniques has given rise to novel diagnostic and treatment methods, including imaging screening tools and predictive applications [33, 34]. The integration of non-invasive diagnostics with AI signifies a paradigm shift in modern medicine, thereby empowering healthcare providers to address health challenges proactively and holistically.

The aim of this study was to develop and validate a Predictive Health Index (PHI) integrating body composition and HRV and ANS variables in order to differentiate between healthy subjects (CTRL) and subjects with cardiovascular, metabolic, psychological, neoplastic, and inflammatory diseases, evaluating the predictive ability of the model in a large clinically characterized sample. The current research thus sought to establish the PHI as a robust, scalable, and practical solution for predictive health monitoring. Moreover, the research explored the integration of AI and non-invasive diagnostic tools into clinical practice. In this process, a series of variables obtained by non-invasive instrumental diagnostic tests of body composition [26–28] and HRV analyses [29–31] were analyzed. We employed AI algorithms [24, 25] to evaluate the weights of these variables in the clinical field and determined their relations to the health status and/or presence of specific pathologies using BIG data of clinically followed patients with the goal of training [14, 15, 35] a PHI that accurately identifies the probability of health maintenance/reserve and/or class of chronic disease development risk.

## Materials and methods

### Study design and sample population distribution

The aim of this study was to validate the PHI for chronic cardiovascular, metabolic, psychological, neoplastic, and inflammatory disorders. Data were obtained from two large multicenter studies employing non-invasive diagnostic technologies for body composition analysis (BIA-ACC^®>^, Biotekna, Marcon, Italy) and HRV analysis (PPG^®^ Stress Flow, Biotekna, Marcon, Italy). Data collection occurred between February 1, 2018, and December 31, 2023, including 35,405 individuals [28, 30].

The study population consisted of 35,405 Caucasian individuals (11,443 men and 23,962 women) aged from 18 to 90 years, with a median age of 54 years. Participants were clustered into one healthy control group (CTRL) and five clinical groups based on chronic disease patient history. Ethical approval for data usage and incorporation of the BIA-ACC^®^ and PPG Stress Flow^®^ devices were ensured by the Ethics Committee of the University Research Institute of Maternal and Child Health and Precision Medicine, National and Kapodistrian University of Athens, Athens, Greece [28, 30]. All procedures adhered to the ethical standards of the Committee Responsible for Human Experimentation and the 1975 Declaration of Helsinki, as revised in 1983, while all apparatuses have been CE-certified as non-invasive medical devices employed for diagnostic and monitoring purposes since 2004.

The CTRL included individuals with no NCDs nor declaring medically unexplained symptoms (MUS). The clinical groups consisted of five distinct clusters, specifically:

- Cardiovascular disorders (referred to as hypertension, atherosclerosis, myocardial infarction, heart failure, cardiac arrhythmia, cardiomyopathies, stroke),
- Metabolic syndrome (referred to as obesity, dyslipidemia, insulin resistance, hyperglycemia, type 2 diabetes mellitus),
- Psychological disorders (referred to as of anxiety or depression).
- Neoplastic disorders.
- Chronic inflammatory disorders (referred to as rheumatoid arthritis, inflammatory bowel disease, ankylosing spondylitis, psoriasis, Crohn’s disease, psoriatic arthritis, Hashimoto’s thyroiditis, celiac disease, or other chronic inflammatory conditions).

**Table 1:** Summarizes the population demographics and dataset split into training and testing subsets. Notably, women were more than twice represented in the dataset when compared to men, highlighting a gender imbalance in the study population (Fig. 1)

Group	Total [*n*]	Train data size [*n*]	Test data size [*n*]	Age [years]
CTRL	2,499	1,874	625	42 [20–66]
CASE	32,906	24,679	8,227	55 [27–76]
Cardiovascular	12,262	9,196	3,066	60 [38–79]
Metabolic	7,474	5,605	1,869	58 [31–77]
Psychological	6,785	5,088	1,697	51 [23–75]
Neoplastic	3,155	2,366	789	57 [37–78]
Inflammatory	14,966	11,224	3,742	54 [27–76]
TOTAL	35,405	26,553	8,852	54 [26–76]

**Fig. 1:**
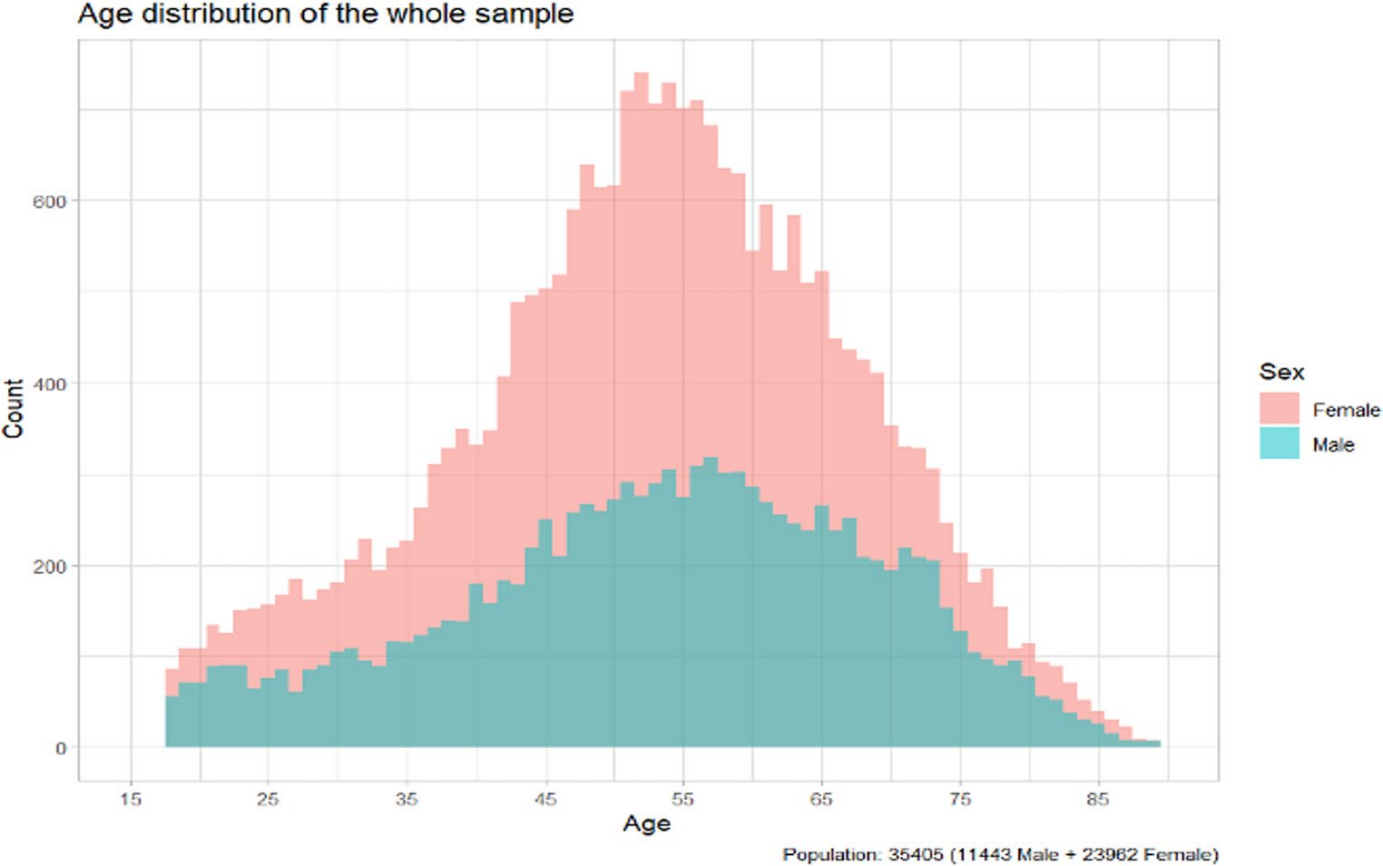
Age distribution of the study population. This population consisted of 11,443 Caucasian men and 23,962 Caucasian women, aged 18 to 90 years (median age: 54 years). Data were extracted from two large multicenter studies using the non-invasive medical devices BIA-ACC and PPG Stress Flow, between 01/02/2018 and 12/31/2023

Table 1. The median [5th-95th percentile] of age, train and test data size in the studied populations stratified by class of NCDs. Summaries are reported as median [5th–95th percentile] for consistency with the original analytic plan; future extensions of the work will provide [2.5th–97.5th].

### Non-invasive devices for data collection

Non-invasive diagnostic devices were employed to collect physiological and health-related data. Dual-frequency bioimpedance measurements were conducted with the BIA-ACC^®^ medical device (BioTekna, Marcon, Italy) for extensive body composition analysis. This device offers an advanced apparatus, as previously described in Peppa et al. [26–28, 30]. The parameters provided were HPA axis index (PA), extracellular water percentage (ECW%), intracellular water percentage (ICW%), total body water percentage (TBW%), T-score, S-score, fat mass percentage (FM%), intramuscular adipose tissue percentage (IMAT%), and extracellular matrix percentage (EC matrix%).

HRV analysis was administrated using the medical device PPG Stress Flow^®^ (BioTekna – Italy) [30], an optoelectronic plethysmograph measuring the change in blood volume for analysis and biofeedback of HRV of the autonomic nervous system measured parameters such as average heart rate (BPM), root mean square of successive differences (RMSSD), standard deviation of NN intervals (SDNN), low-frequency/very low-frequency ratio (LF/VLF), and total power.

Table 2. All parameters used to validate the PHI.

**Table 2:** Includes the parameters employed to calculate the PHI

Age	Years	Represents the age of the individual
Sex	Categorical	Indicates the gender of the individual, typically labeled as male or female
PA (PA axis index)	Degrees **(°)**	Phase angle (PA) is a measure obtained through bioimpedance analysis It is a parameter that reflects the integrity of cell membranes and represents the activity index related to the hypothalamic-pituitary-adrenal axis - HPA axis index
ECW %	Percentage	Represents the percentage of extracellular water relative to total body water
ICW%	Percentage	Represents the percentage of intracellular water relative to total body water, obtained through bioimpedance
TBW%	Percentage	Represents the percentage of total body water
T-score	Standardized score	Indicates bone density, compared to a healthy 30-year-old adult of the same sex
S-score	Standardized score	Represents the relative muscle mass compared to a healthy 30-year-old adult of the same sex
FM %	Percentage	Represents the percentage of fat mass relative to total body mass
IMAT%	Percentage	Represents the percentage of intramuscular adipose tissue relative to total body mass
EC Matrix%	Percentage	Represents the percentage of extracellular matrix relative to total body mass
BPM (HR)	Beats per minute	Represents the heart rate, typically measured as part of heart rate variability (HRV) analysis
RMSSD	Milliseconds	Represents the root mean square of successive differences between normal heartbeats, an HRV measure indicating parasympathetic activity
SDNN	Milliseconds	Represents the standard deviation of NN intervals (the intervals between normal heartbeats), an HRV measure indicating overall autonomic balance
LF/VLF	Ratio	Represents the ratio of low frequency to very low frequency components in HRV, used to analyze autonomic nervous system balance
Total power	Milliseconds squared	Represents the total power of all frequency components in HRV, indicative of overall autonomic nervous system activity

### Statistical analyses

#### Statistical analysis and machine learning model

Analyses were performed in R (v. 4.2.2, The R Foundation for Statistical Computing). Continuous variables are presented, unless otherwise specified, as median [5th–95th percentile]; categorical variables as n (%). Statistical significance was set at *p* < 0.05. To construct the PHI, five independent binary classification random forest models (one for each clinical category compared to CTRL) were trained using the *randomForest* package (Liaw & Wiener, 2002). No hyperparameter optimization was performed; the model was therefore trained with the package default settings. Variable importance was assessed using mean decrease in Gini impurity. The dataset was divided via a 75%/25% stratified random split at the subject level (Training/Test), maintaining proportions between groups. Validation included the Mann-Whitney U test on predicted score distributions, ROC curves, and AUC (area under the ROC curve). The probability of disease was estimated based on predicted values using a generalized additive model (GAM). No 95% confidence intervals for AUC were calculated in this version; such an analysis is planned for future methodological extension.

The PHI was assessed by means of statistical and ML methods aiming to obtain valuable results and validate the predictive performance, focusing on the use of random forest models to predict health outcomes based on specific physiological parameters.

**Step-by-step description**

1. **Dataset preparation and loading**
  ·Load the complete dataset containing both CTRL and CASE data.
  ·Randomly split the dataset into two parts:
  ·**Trainingset** (75% of the CTRL dataset + 75% of the CASE dataset)
  ·**Testset** (25% of the remaining CTRL dataset + 25% of the remaining CASE dataset)
2. **Random forest algorithm model training**
   ·Use the “training set” to train the random forest algorithm:
  ·Input: PPG Stress Flow + BIA-ACC® parameters
  ·Output: predicted value: probability of presence or absence of pathology/disorder
3. **Validation metrics**
   ·Validate the trained random forest model employing the test set using:

#### Dataset preparation and loading

Comprisin***g*** both CTRL and case (CASE) data, the complete dataset was merged and randomly split into two parts, as follows: (a) training set (75% of the CTRL dataset + 75% of the CASE dataset) and (b) test set (25% of the remaining CTRL dataset + 25% of the remaining CASE dataset) operational accuracy of the model and to ensure that all clinical categories remain proportionally represented.

Handling of missing data: Main analyses were conducted using complete-case analysis: records missing one or more required parameters were excluded during pre-processing. No imputation of missing data was performed. This choice is discussed among the limitations.

#### Model training

The training set was employed to train a random forest algorithm using as input the features derived from the BIA-ACC^®^ and PPG Stress Flow^®>^ metrics. The target variable was identified and set, referring to the presence or absence of pathology. Next, a random forest classifier is initialized using a machine learning library [35, 36]. Training is initiated by running the model with the training set, using the defined features and target variable. This training enables the random forest algorithm to identify patterns and feature relations that influence predicting pathology. In addition to the single 75/25 stratified train–test split, we implemented 10-fold cross-validation to further assess the robustness and generalizability of the random forest models. Cross-validation was conducted at the subject level to prevent data leakage, with folds stratified to preserve the proportion of cases and controls within each clinical category. Performance metrics from each fold were averaged to obtain mean estimates with 95% confidence intervals, which are reported alongside the test-set results.

#### Validation metrics

In this step, the trained random forest model obtained using the test set through various statistical and graphical methods. For all analyses, a p-value of 0.05 was considered statistically significant, while bioinformatic analyses were performed using R software (version 4.2.2, The R Foundation for Statistical Computing). The probability of disease is estimated based on random forest predicted values using a GAM. Model performance was quantified using a comprehensive set of metrics: accuracy, sensitivity, specificity, positive predictive value (PPV), negative predictive value (NPV), F1-score, and AUC. Confusion matrices were also derived for each clinical category. To provide robust estimates, 95% confidence intervals were computed for all metrics, including AUC, via stratified bootstrapping with 1000 resamples. ROC curves with 95% confidence bands were plotted for each model. Variable importance was assessed using the mean decrease in Gini impurity, as implemented in the randomForest package, and features were ranked accordingly.

Furthermore, a box-whisker plot is generated to visually represent the distribution of the predicted probabilities, thereby providing a graphical representation of the data spread. The disorder probability is calculated by fitting the disorder with predicted values obtained with the random forest method using a generalized additive model (GAM) [37]. GAM can be considered a generalized linear model (GLM), in which the linear predictor is given by a user-specified sum of smooth functions of the covariates, plus a conventional parametric component of the linear predictor.

Additionally, the ROC curve metric is plotted to evaluate the model’s performance in terms of sensitivity and specificity, illustrating the trade-off between true and false positive rates. Finally, the AUC value is calculated to quantify predictive accuracy, with a higher AUC indicating better model performance. These validation steps ensure that the model’s predictions are reliable and accurate [38].

For each clinical category, specific statistical tests were conducted and the model’s performance metrics were calculated to evaluate the effectiveness of the random forest in predicting the presence or absence of disorders and diseases [Predictive Health Index, PHI]. Additionally, key predictive variables were derived from the analysis. Variable importance identified the most influential features per case, including age, HPA axis index, and specific bioimpedance and HRV metrics providing an interpretation of their role in the context of the analyzed disorders. In this light, predicted probability distribution was conducted presenting the correlation between the model’s predicted values and the associated probabilities are examined, highlighting the linearity of the relations.

##### Outcome definition

For each of the five clinical categories, independent binary models (CASE vs. CTRL) were trained. No multi-label classification was implemented.

## Results

### Mann-whitney u test

**Fig. 2:**
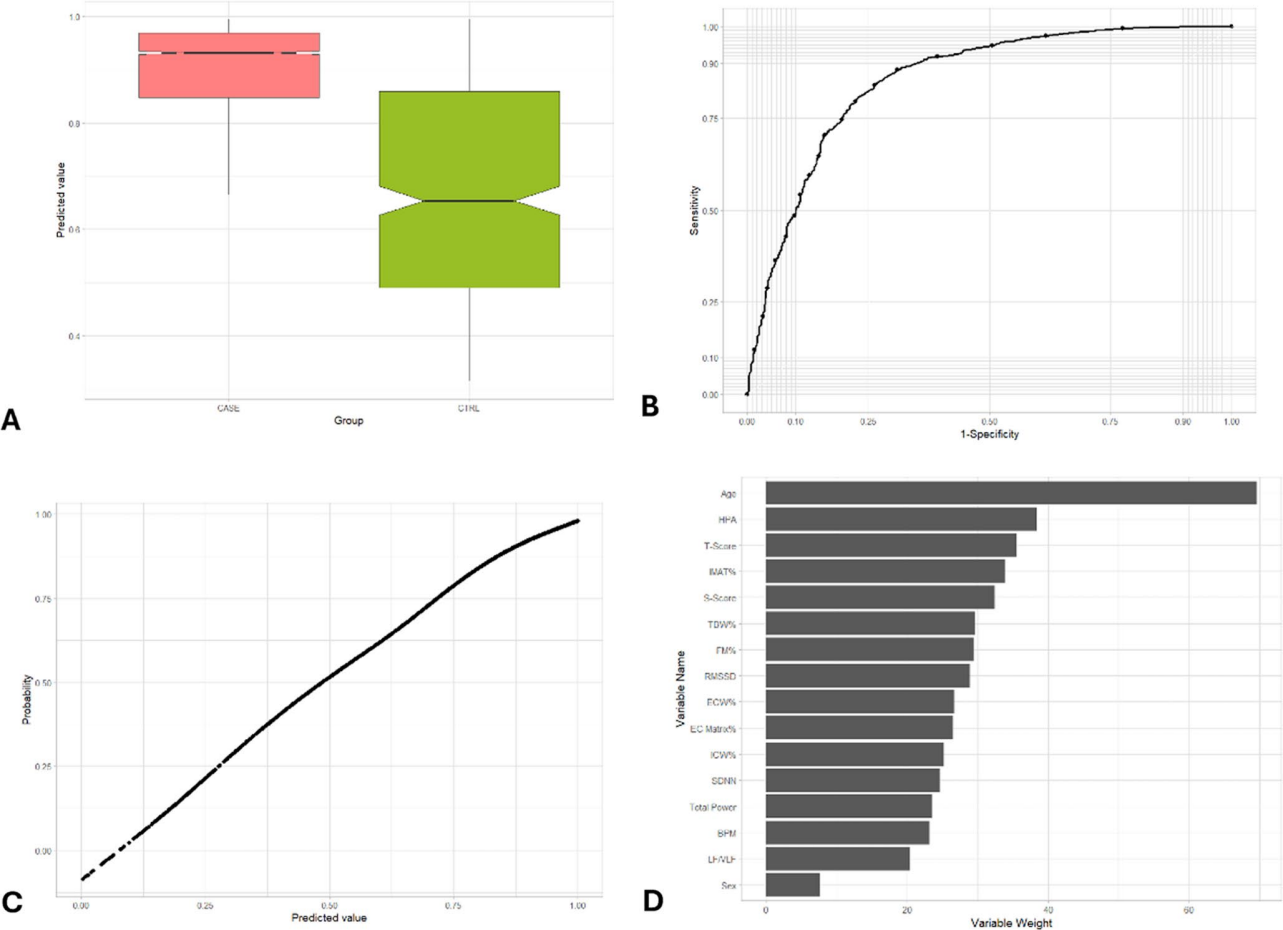
Representation of the main metrics and tests used to validate the prediction model for the identification of cardiovascular disorders/diseases. (**A**) Box-whisker plot of predicted value in subjects with cardiovascular disorders (CASE) vs. CTRL group. The box shows the interquartile range (IQR). A solid line connects the observations within 1.5 interquartile ranges. Solid black circles identify the outliers. Notch displays the 95% confidence interval around the median which is normally based on the median +/- 1.57 x IQR/sqrt of n. (**B**) ROC curve of predicted value in subjects with cardiovascular disorders (CASE) vs. CTRL group. AUC = 0.853. (**C**) Cardiovascular disorders/diseases probability based on predicted value. (**D**) Variables weight on random forest machine learning algorithm

**Fig. 3:**
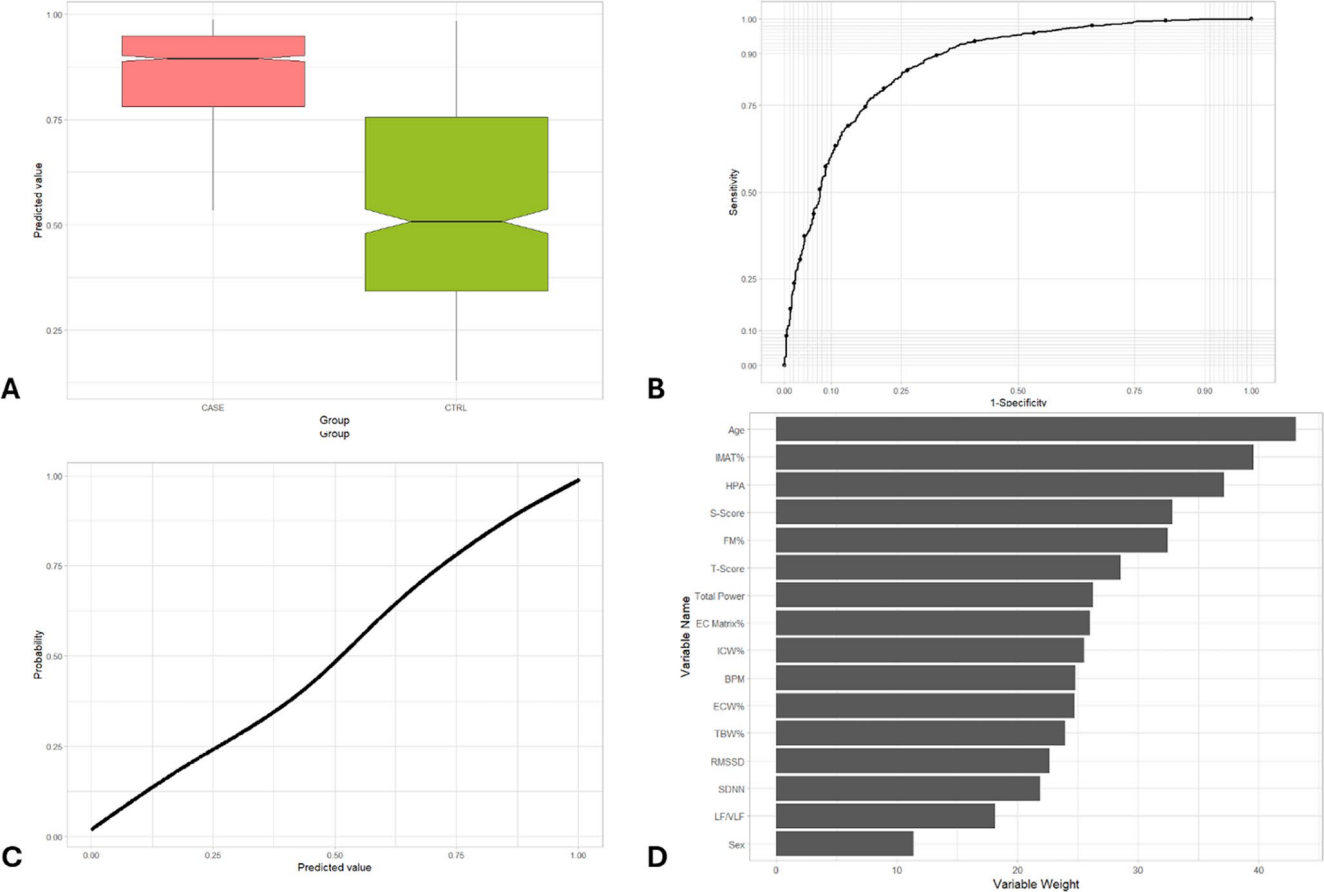
Representation of the main metrics and tests used to validate the prediction model for the identification of metabolic syndrome.(**A**) Box-whisker plot of predicted value in subjects with metabolic syndrome (CASE) vs CTRL group. The box shows the interquartile range (IQR). A solid line connects the observations within 1.5 interquartile ranges. Solid black circles identify the outliers. Notch displays the 95% confidence interval around the median which is normally based on the median +/- 1.57 x IQR/sqrt of n. (**B**) ROC curve of predicted value in subjects with metabolic syndrome (CASE) vs. CTRL group. AUC = 0.870. (**C**) Metabolic syndrome probability based on predicted value. (**D**) Variables weight on random forest machine learning algorithm

**Fig. 4:**
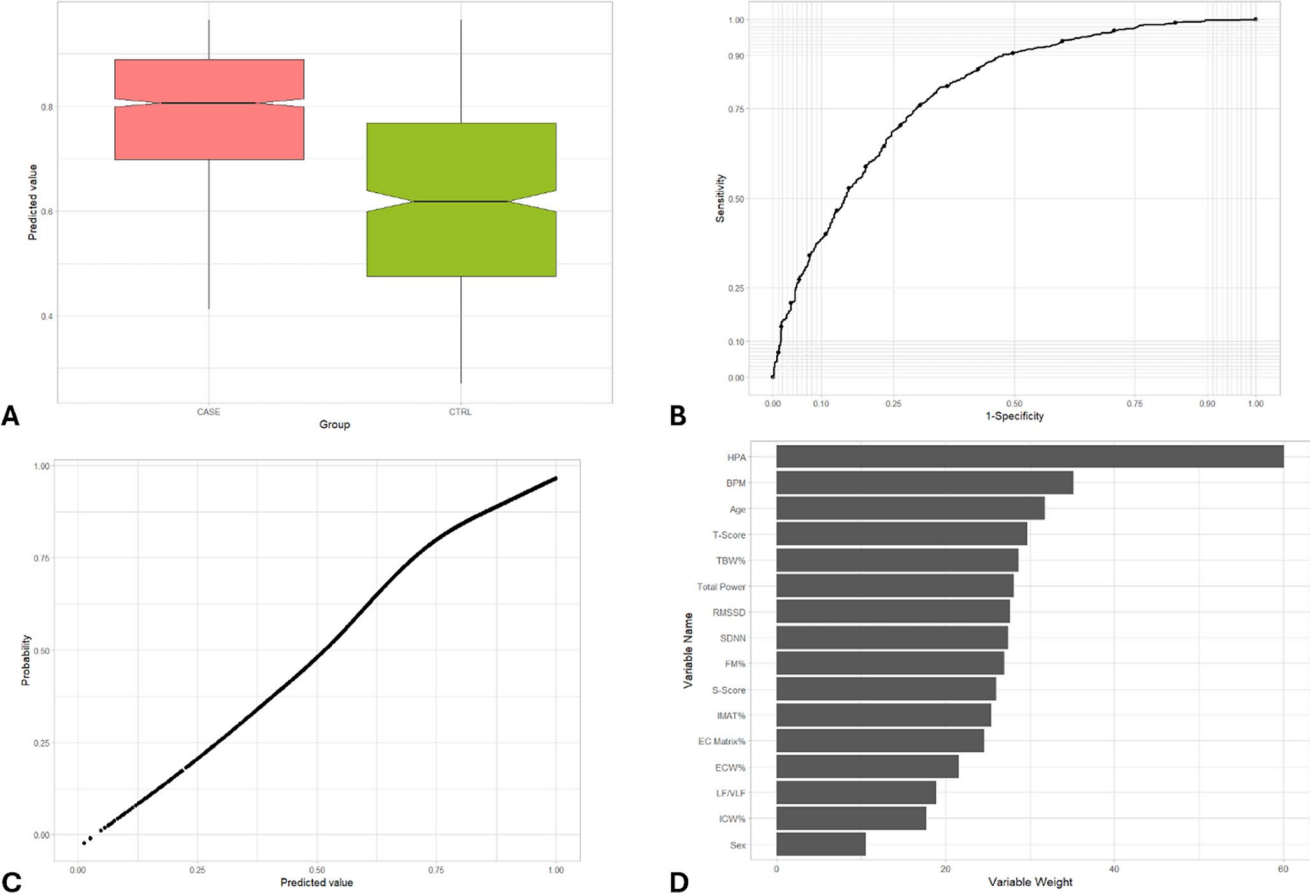
Representation of the main metrics and tests used to validate the prediction model for the identification of psychological disorders. (**A**) Box-whisker plot of predicted value in subjects with psychological disorders (CASE) vs. CTRL group. The box shows the interquartile range (IQR). A solid line connects the observations within 1.5 interquartile ranges. Solid black circles identify the outliers. Notch displays the 95% confidence interval around the median which is normally based on the median +/- 1.57 x IQR/sqrt of n. (**B**) ROC curve of predicted value in subjects with psychological disorders (CASE) vs. CTRL group. AUC = 0.794. (**C**) Psychological disorders probability based on predicted value. (**D**) Variables weight on random forest machine learning algorithm

**Fig. 5:**
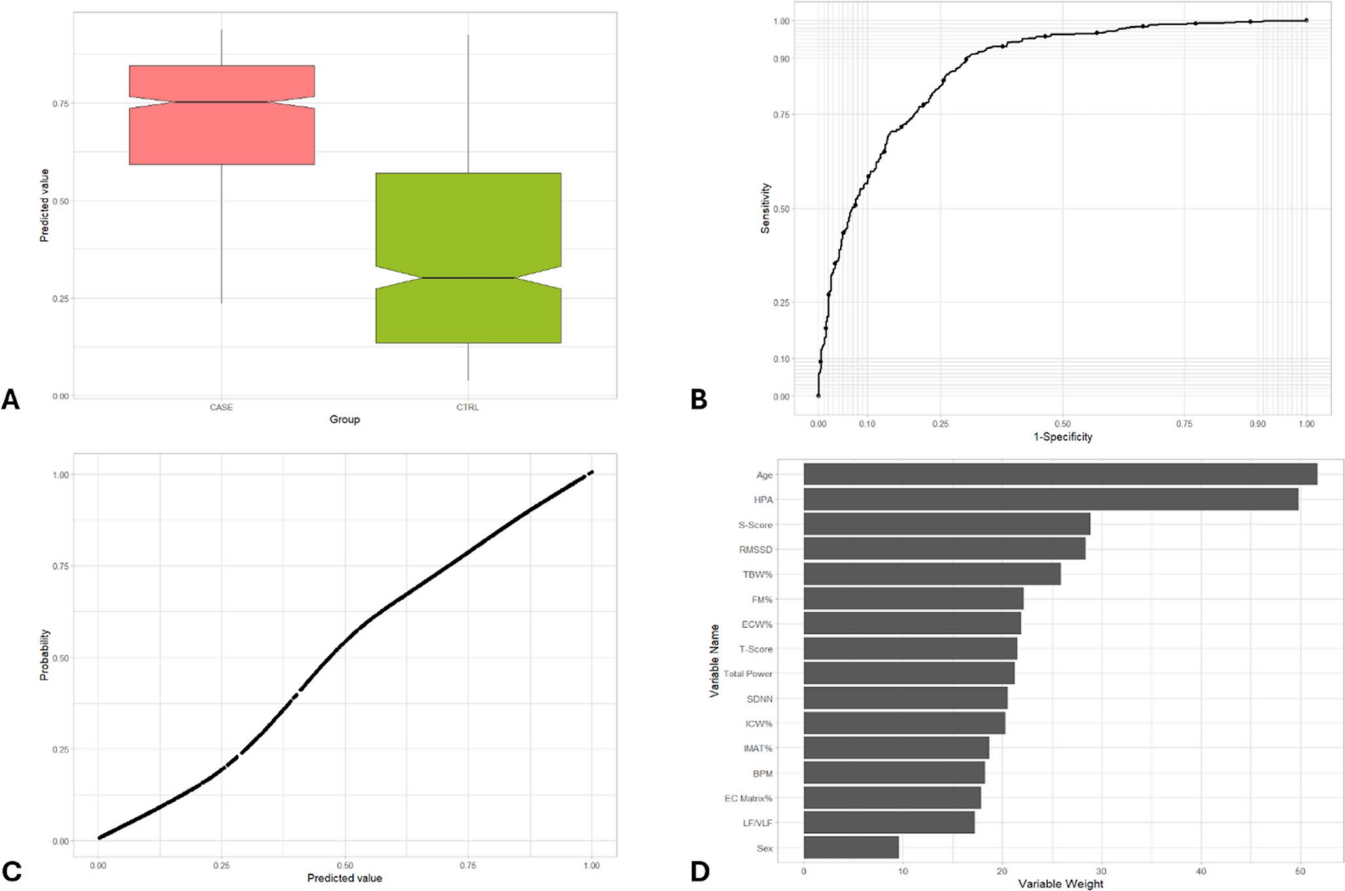
Representation of the main metrics and tests used to validate the prediction model for the identification of neoplastic disorders. (**A**) Box-whisker plot of predicted value in subjects with cancer (CASE) vs. CTRL group. The box shows the interquartile range (IQR). A solid line connects the observations within 1.5 interquartile ranges. Solid black circles identify the outliers. Notch displays the 95% confidence interval around the median which is normally based on the median +/- 1.57 x IQR/sqrt of n. (**B**) ROC curve of predicted value in subjects with cancer (CASE) vs. CTRL group. AUC = 0.871. (**C**) Cancer probability based on predicted value. (**D**) Variables weight on random forest machine learning algorithm

The Mann-Whitney U test was conducted to compare the distributions of the predicted probabilities for the presence and absence of psychological, neoplastic, and chronic inflammation disorders and metabolic syndrome in the test set. The p-value obtained from this test is less than 0.001 in all five pathological cases (Table 3; Fig. 2A,3 A,4 A,5 A,6 A). This extremely low p-value indicates a statistically significant difference between the predicted probability distributions of the two groups (Control group and Case group) for each condition. In other words, the Random Forest model can discriminate between individuals who have these specific illnesses and those who do not. The considerable difference indicates that the model’s predictions are not random but rather reflect meaningful patterns in the data.

**Table 3:** Performance characteristics of an AI-based non-invasive predictive health index

	*P*-value (Mann-Whitney U test)	AUC	Most influential variables
Cardiovascular disorders	< 0.001	0.853	Age, PA axis index, T-score
Metabolic syndrome	< 0.001	0.870	Age, percentage of IMAT, PA axis index
Psychological disorders	< 0.001	0.794	PA axis index, heart rate, age
Neoplastic disorders	< 0.001	0.871	Age, PA axis index, S-score
Chronic inflammatory disorders	< 0.001	0.778	Age, PA axis index, RMSSD

**Fig. 6:**
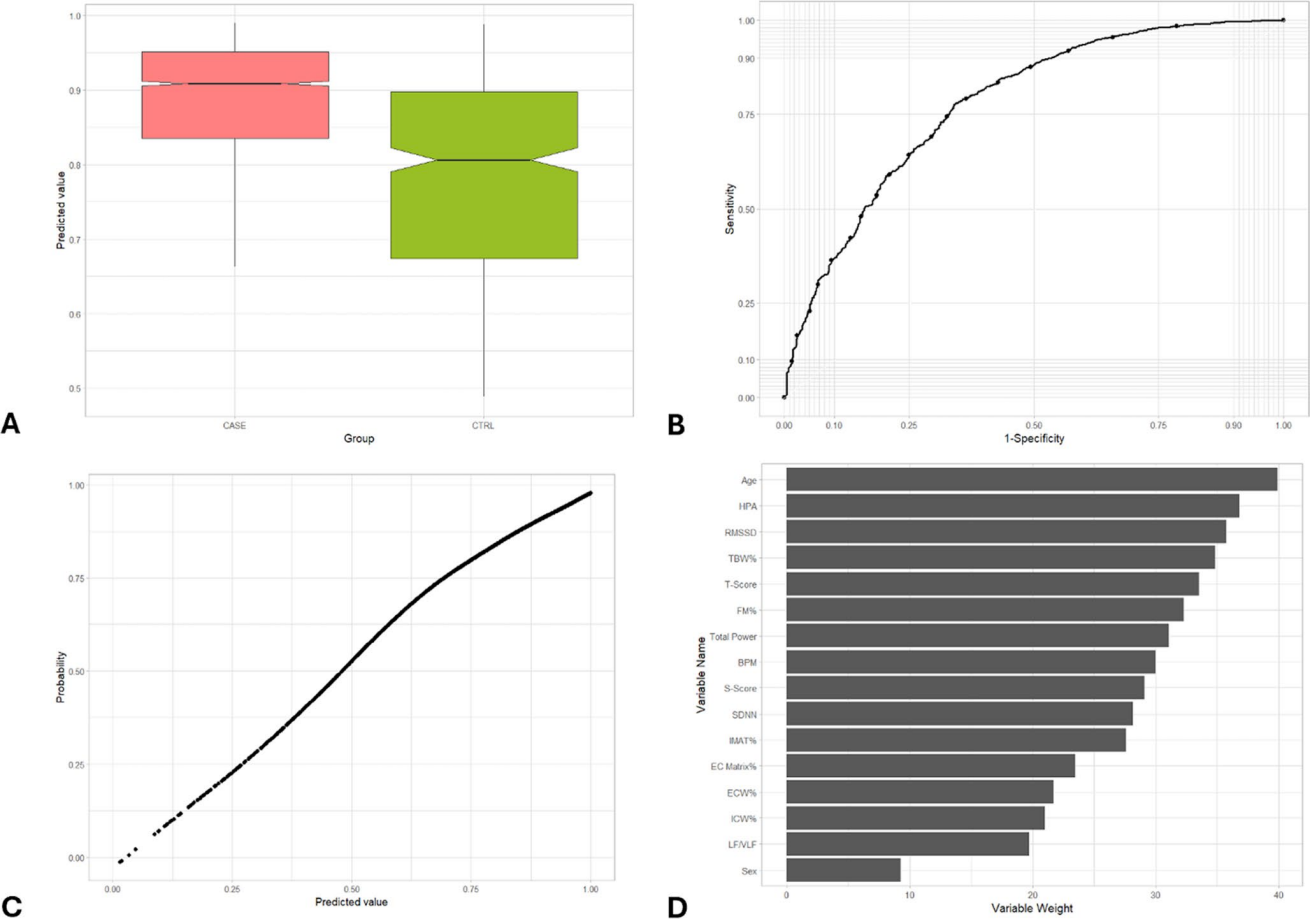
Representation of the main metrics and tests used to validate the prediction model for the identification of chronic inflammation disorders. (**A**) Box-whisker plot of predicted value in subjects with chronic inflammation disorders (CASE) vs. CTRL group. The box shows the interquartile range (IQR). A solid line connects the observations within 1.5 interquartile ranges. Solid black circles identify the outliers. Notch displays the 95% confidence interval around the median which is normally based on the median +/- 1.57 x IQR/sqrt of n**. **(**B**) ROC curve of predicted value in subjects with chronic inflammation disorders (CASE) vs. CTRL group. AUC = 0.778.(**C**) Chronic inflammation disorders probability

### AUC

The AUC is a measure of the model’s ability to discriminate between the positive class (presence of the disorder) and the negative class (absence of the disorder), whereas an AUC of 1.0 represents perfect discrimination. The AUC values for each condition are 0.853 (cardiovascular), 0.870 (metabolic), 0.794 (psychological), 0.871 (neoplastic), and 0.778 (inflammatory), indicating a moderate to good discriminative ability of the model. (Table 3; Figs. 2B, 3B, 4B, 5B and 6B). More specifically, as for cardiovascular disorders, metabolic syndrome, psychological, neoplastic and chronic inflammatory diseases, AUC is 0.853, 0.870, 0.794, 0.871, and 0.778, respectively. An AUC of this excellence means that the model has a strong ability to correctly classify individuals who exhibit or lack each pathology.

### Probability vs. predicted value

The model’s predicted values, which are numerical outputs ranging from 0 to 1, exhibited a monotonic relation between predicted value and estimated probability of the condition (Fig. 2 C, 3 C, 4 C, 5 C, 6 C). The relation was estimated using a GAM. As the values increased, the probability of the presence of each ailment rose proportionally and consistently. This relation highlights the robustness of the model in translating predicted values into probabilities, providing an interpretable and reliable framework for assessing the likelihood of these five illnesses..

### Variable importance

Beyond performance metrics, an essential aspect of the random forest model is identifying the key variables that contribute most significantly to its predictions. For cardiovascular disorders, the three most impactful variables in the model are age, HPA axis index, and T-score (Table 3; Fig. 2D). These variables play a pivotal role in shaping the model’s predictions, with age standing out as a well-established risk factor for cardiovascular diseases, highlighting the increased vulnerability associated with aging. Regarding metabolic syndrome, age, percentage of IMAT, and HPA axis index comprise the three variables with the most weight in the random forest algorithm for predicting this case of illness (Table 3; Fig. 3D), while the most important variables in psychological disorders include HPA axis index, heart rate and age (Table 3; Fig. 4D). Moreover, the three variables with the most weight in the random forest algorithm for predicting cancer are age, HPA axis index, and S-score (Table 3; Fig. 5D), while as for chronic inflammatory disorders, the factors which have been identified as the most influential in determining the model’s output is age, HPA axis index, and RMSSD (Table 3; Fig. 6D). The random forest variable importance analysis revealed consistent predictors across categories. Features were ranked by mean decrease in Gini impurity, with age and HPA axis index emerging as dominant contributors in all five models. Additional high-ranking features included T-score (cardiovascular disorders), percentage of IMAT (metabolic syndrome), heart rate (psychological disorders), S-score (neoplastic disorders), and RMSSD (inflammatory disorders). Ranked feature importance tables for each category are provided in Supplementary Table S1. These results support the biological plausibility of the PHI, aligning with established risk factors such as age and stress-related neuroendocrine activity.

## Discussion

Predictive medicine represents a crucial advancement in the ability to prevent diseases using a proactive approach aimed at improving overall individual and societal health. This study validated a PHI based on non-invasive integration of bioimpedance (BIA-ACC^®^) and HRV (PPG Stress Flow^®^) via random forest, with moderate to good discriminative performance across five clinical categories. The PHI is designed to be a rapid, non-invasive, and scalable tool for health status and disease risk assessment. From this particular perspective, the PHI concept is potentially extendable to other devices measuring analogous variables with dedicated calibration and validation; the present validation applies exclusively to the devices used in this work.

The results showed high significance (p-value < 0.0001) of the PHI, thus demonstrating a highly accurate predictive capability. The model’s accuracy, reflected in high AUC values (ranging from 0.778 to 0.871 across disease categories), underscores its effectiveness as a real time diagnostic tool. Key metrics used for model validation included the Mann-Whitney U test and the AUC of the ROC curves, all revealing a promising development of our main concept. Our overall results hence indicated that an AI-based predictive non-invasive approach is highly effective in forecasting the health status and disease risk of an individual, confirming the usefulness of the random forest model for predictive healthcare. This tool can become fundamental in modern medicine, which is shifting from a reactive to a proactive approach to health maintenance, thereby improving quality of life and reducing suffering and overall healthcare costs.

An important aspect concerns age, one of the most influential variables in the models. Differences in age between the clinical groups and the younger CTRL group represent a potential source of confounding, even though age was included among predictive features. Future studies should harmonize age between groups or apply stricter matching/adjustment strategies to better isolate the effects of body composition and HRV variables.

In the group with psychological disorders, there is a greater overlap with CTRL. This may reflect clinical and severity heterogeneity, the presence of subclinical phenotypes, as well as intra-subject variability of HRV measures and HPA axis dynamics. The future integration of additional biomarkers and longitudinal assessment could improve separation.

Another important methodological consideration concerns the handling of missing data. We employed a complete-case analysis, excluding records with missing values. While this ensured internal consistency and avoided bias from imputation assumptions, it may reduce generalizability by preferentially including individuals with more complete datasets. Missingness may not be random, and therefore our results could under-represent participants with higher comorbidity or lower data quality. Future studies will address this limitation by applying multiple imputation or model-based approaches to better preserve sample representativeness and improve robustness.

Considering the findings of this study, it is recommended that clinical implementation and integration of AI-based predictive models into health assessment protocols be explored. Despite the promising results, several limitations must be acknowledged. Education and awareness should be enhanced among physicians and the public regarding the value of proactive health monitoring. The dataset used was derived from a predominantly Caucasian population, which may limit the generalizability of the findings, underscoring the need for validation across more diverse demographic and geographic groups. Moreover, while the random forest model demonstrated highly predictive accuracy, interpretability remains a challenge for complex machine learning models. Long-term testing, continuous data registration for algorithm retraining, and benchmarking against other models will be required to ensure robustness and reliability. Future research should also incorporate explainable AI approaches to enhance transparency and trust in predictions, expand the PHI to include broader biomarker panels, and test its performance in longitudinal studies. Integrating genetic, epigenetic, and lifestyle data could further strengthen its predictive capacity and relevance. Equally important will be the development of user-friendly applications and interfaces to facilitate implementation in both clinical and community settings.

This study establishes the PHI as a novel and effective tool for proactive health management. The shift toward non-invasive diagnostics and AI-driven platforms provides a comprehensive means of assessing health status and predicting disease risk. By enabling early detection, guiding personalized interventions, and supporting preventive strategies, the PHI represents a step forward in addressing the rising burden of chronic disease. Continued refinement, validation, and clinical integration of this tool will be critical in promoting its role within modern healthcare and contributing to a more proactive and sustainable health system.

Looking ahead, the future of AI-enabled devices in personalized medicine is extremely promising. Advances in wearable biosensors, edge computing, and federated learning will make it possible to collect, analyze, and interpret health data streams in real time while maintaining privacy and security. When coupled with genomic, epigenomic, and lifestyle information, AI will move beyond population-level predictions to deliver truly individualized health insights. This convergence will empower patients to take an active role in their own health while equipping clinicians with data-driven tools for precise and timely decision-making. As AI-enabled devices mature, they are expected to become foundational to precision medicine, creating a healthcare paradigm centered on continuous monitoring, tailored prevention, and targeted intervention — ultimately reshaping the delivery of care for the decades ahead.

## Abbreviations

PHI: Predictive Health Index
NCDs: Non-communicable diseases
HRV: Heart rate variability
PPG: Photoplethysmography
AI: Artificial intelligence
ML: Machine learning
DL: Deep learning
BIA: Bioimpedance analysis
CTRL: Control group
